# Epidemiological surveillance of tegumentary leishmaniasis: local territorial analysis

**DOI:** 10.1590/S1518-8787.2017051006614

**Published:** 2017-06-20

**Authors:** Valdenir Bandeira Soares, Andréa Sobral de Almeida, Paulo Chagastelles Sabroza, Waldemir Paixão Vargas

**Affiliations:** I Departamento de Endemias Samuel Pessoa. Escola Nacional de Saúde Pública. Fundação Oswaldo Cruz. Rio de Janeiro, RJ, Brasil

**Keywords:** Leishmaniasis, Cutaneous, epidemiology, Geographical Localization of Risk, Geographic Mapping, Epidemiological Surveillance

## Abstract

**OBJECTIVE:**

To propose a new operational unit in the locality scale capable of subsidizing the construction of an information system to control the transmission of tegumentary leishmaniasis at this scale, in a region of high endemicity of the Atlantic Forest.

**METHODS:**

We examined the adequacy of data and instruments in an area of high endemicity in the Atlantic Forest located in the South of the State of Rio de Janeiro from 1990 to 2012. The study proposed an operational unit called Local Surveillance Unit to make all used databases compatible by adjusting census sectors. This enabled the overlap and comparison of information in different periods.

**RESULTS:**

The spreading process of the transmission of tegumentary leishmaniasis in the Baía da Ilha Grande region does not depend on great population movements, and can occur in areas with population growth or decrease. The data information system allowed the adequate identification and characterization of the place of residence. We identified relevant characteristics of the place of transmission, such as self-limited in time and not associated with recent deforestation. The results also highlight the lack of synchronicity in the case production in territorial units involved in the endemic-epidemic process, noting that this process is in constant motion.

**CONCLUSIONS:**

The transmission process seems more connected to the presence and movement of rodents that move continuously in the region than to the local density of vectors or the permanence of infected dogs at home. New control strategies targeted at the foci of transmission must be considered. The construction of a new operational unit, called Local Surveillance Unit, was instrumental in the endemic-epidemic process analysis.

## RESUMO

**OBJETIVO:**

Propor uma nova unidade operacional na escala de localidade capaz de subsidiar a construção de um sistema de informação orientado para o controle da transmissão da leishmaniose tegumentar nesse nível.

**MÉTODOS:**

Uma região de alta endemicidade da Mata Atlântica no sul do estado do Rio de Janeiro de 1990 a 2012 foi selecionada para analisar a adequação dos dados e instrumentos. Uma unidade operacional denominada Unidade de Vigilância Local foi proposta para compatibilizar todos os bancos de dados utilizados por meio de ajustes dos setores censitários. Isso possibilitou a sobreposição das informações e a sua comparação em diferentes períodos.

**RESULTADOS:**

O processo de deslocamento da transmissão da leishmaniose tegumentar na região da Baía da Ilha Grande não dependeu de movimentos populacionais importantes, podendo ocorrer tanto em áreas com crescimento como em áreas com decremento populacional. Os dados do sistema de informação permitiram a identificação e caracterização adequada do local de residência. Identificaram-se características relevantes do lugar de transmissão, como autolimitados no tempo e não associados a desmatamentos recentes. Os resultados evidenciam também a falta de sincronicidade na produção de casos nas unidades territoriais envolvidas no processo endêmico-epidêmico, mostrando que esse processo está em constante movimento.

**CONCLUSÕES:**

O processo de transmissão parece estar mais ligado à presença e circulação de roedores que se desloquem continuadamente na região do que da densidade local de vetores ou da permanência de cães infectados no domicílio. Novas estratégias de controle orientadas para os focos de transmissão devem ser consideradas. A construção de uma nova unidade operacional, denominada Unidade de Vigilância Local, foi fundamental na análise do processo endêmico-epidêmico.

## INTRODUCTION

Brazil has seen a sharp increase in cases of tegumentary leishmaniasis (TL) in the last decades^13,14^. At the same time, the occurrence of different epidemiological patterns of this endemic disease in different geographic regions has also been noted. In the Southeast, the pattern is of type II, with old colonization and the presence of residual forests, and transmission occurs mainly at home or peridomestic habitats^a^.

An outbreak investigation in Rio de Janeiro, RJ, in 1974 recorded the same epidemiological characteristics described in foci studies with transmission patterns at home and the surrounding areas. The investigation also enabled the identification of other behavior of this endemic disease: the simultaneous production of discontinuous outbreaks, although as a true network of interrelated foci, and their tendency to dissemination through the progressive incorporation of new locales to the endemic area with active transmission^10,b^.

Until 1996, TL was not as a problem-oriented to surveillance. The information component of the National Program of Tegumentary Leishmaniasis Control was the production of standardized reports with operational indicators, which consolidated the data only per State. The program had as main objectives the early diagnosis with proper treatment of human cases and the reduction of human-vector contact by the application of insecticide and measures of individual protection^12^.

Since then, a restructuring of the TL control program’s information system occurred by agreement between the National Center for Epidemiology’s Technical Management of Vector-borne Diseases and the National School of Public Health, specifically the Department of Endemic Diseases Samuel Pessoa’s Endemic diseases Monitoring Laboratory. The guidance was also considered for surveilling and monitoring of this endemic disease and the municipality was the territorial unit of reference for data consolidation^c^.

Among the main objectives of the restructured program’s surveillance component was assessing the feasibility of building a database on the locality, in order to guide control actions directed reduce new cases and the risk of transmission of TL^c^.

This study’s objective was to propose a new operational unit in the locality scale capable of subsidizing the construction of an information system to control the transmission of TL at that scale, in a region of high endemicity of the Atlantic Forest.

## METHODS

Ecological study based on the development of a new spatial unit for healthy analysis at the local level in an area of high endemicity in the Atlantic Forest located in the South of the State of Rio de Janeiro from 1990 to 2012.

The region of Baía da Ilha Grande is located on the Southern coast of the State of Rio de Janeiro. The region is composed of the municipalities of Angra dos Reis and Paraty. In this area, Serra do Mar offers relief with variable and rugged altitude.

The climate varies from hot to a mild oceanic climate with high rainfall, around 1,500 to 2,000 mm annually. The predominant vegetation is dense ombrophilous forest, be it the primitive Atlantic Forest or the woods of secondary formation in regenerating areas^19^.

Among the major projects deployed in the Baía da Ilha Grande region since the 1950’s is the opening of the Rio-Santos Highway BR-101 in 1973, mainly responsible for social and environmental transformations^d^.

The population was 56,210 inhabitants in 1970 and had increased 368.0% in 2010. This population growth and real estate speculation generated by tourism development forced the population excluded from that process to occupy, in an unorganized manner, mainly the outskirts of urban areas^e,f^.

The recording TL cases was obtained as follows: from 1990 to 1995 we used the Supervision of Public Health Campaign’s (SUCAM) database; from 1996 to 1999 we used an individual database of the National Health Foundation’s (FUNASA) control program’s coordination and from 2000 to 2012 the data incorporated into the database derived from the Information System of Reportable Diseases (SINAN).

With the intent of analyzing TL’s characteristics at a sub-municipal level through the sources of information utilized, we developed a digital graphical base that allows us to overlap information and compare it in different periods.

Localities of the study area were geotagged in a central point of their location. To get this network of points, there were two methods:

- For the city of Angra dos Reis, with a Strategic Plan, a geotagged territorial base already existed in the city, representing each of the locales. The TerraView 4.2.2 application set the center point of each locality by the polygon centroid. To adjust these centroids to the points of greater anthropic pressure in the locality for the year 2002 we used an image of the sensor Landsat 7 ETM+, 5.4 and 3 bands, orthorectified.
- For the city of Paraty, without a Strategic Plan, geographical coordinates of each location’s population centers were taken using the Global Positioning System (GPS). The satellite images helped adjust these coordinates where necessary.

The network of locations used to analyze the characteristics of TL at sub-municipal level was not suitable for analysis of population data. That is because IBGE (Brazilian Institute of Geography and Statistics) only offers its data through rural and urban census sectors (CS), which often do not correspond to the locations of each municipality. To overcome this problem, the Local Surveillance Units^g^ (LSU) have been proposed and their boundaries demarcated by using the application TerraView 4.2.2.

We carried out adjustment of the territorial basis of the urban and rural census sector of 1991^h^ with the territorial basis of the 2000 census^i^. The altitude of 500 m was the level quota used to trim this adjusted territorial base. This was the area of greater human occupation was separated from the one with the rarefied occupation, represented mainly by environmental preservation areas on the slopes of Serra da Bocaina. These census sectors’ resultant layer was overlaid with another layer of localities network. We named each sector after the most representative locale, usually the most populous. The LSU-level databases were the sum of all variables of the various local-level databases.

**Figure 1:**
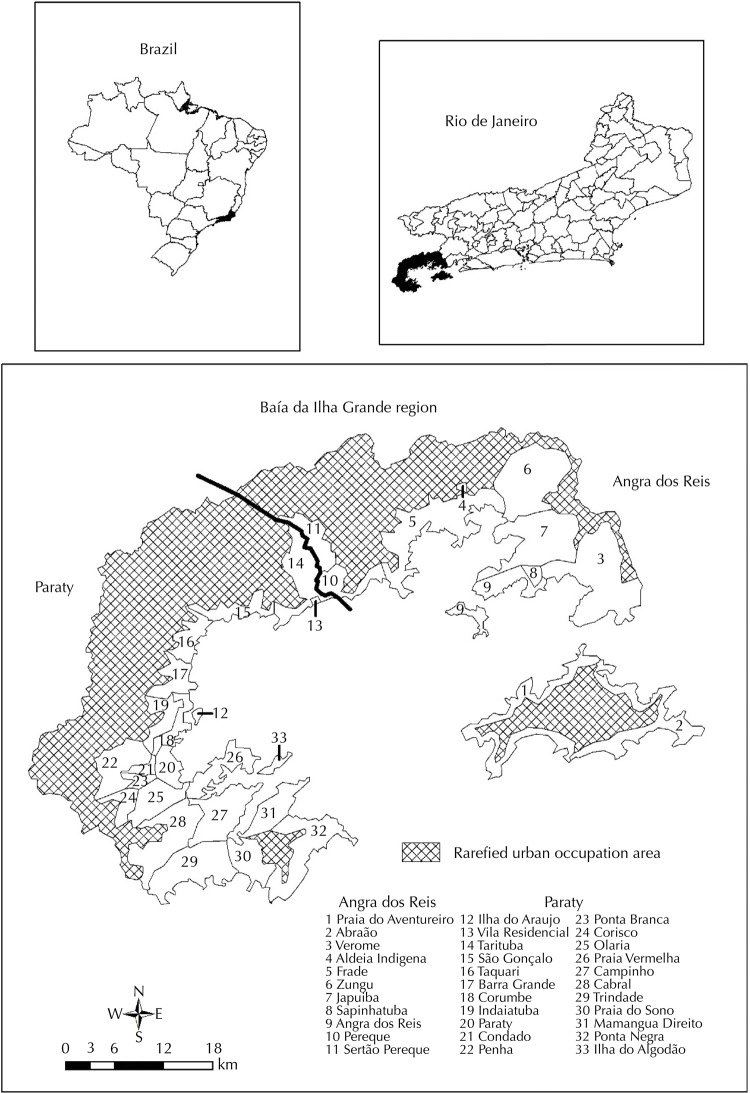
Location map of the Baía da Ilha Grande region per local surveillance unit.

We identified 33 LSU in the Baía da Ilha Grande region after making all databases compatible (Figure 1).

We used a chart with the annual distribution of cases from 1990 to 2012 to identify the fluctuations in the occurrence of TL cases in each endemic region. We straightened the curve through changing averages to mitigate those variations, allowing us to define periods according to the magnitude of the occurrence of TL.

We created one table per LSU to define those more important in the transmission intensity and to observe the stability of cases using liquid density. Liquid density is the number of cases divided by building areas (km^2^) and areas occupied by the LSU’s human population and multiplied by 10. These areas were identified by the classification of the Landsat 7 ETM+’s satellite image from 2002.

**Table:** Characteristics of tegumentary leishmaniasis cases per local surveillance unit. Baía da Ilha Grande region, State of Rio de Janeiro, Brazil, from 1990 to 2012.

Local surveillance unit	Cases from 1990 to 2012	Period 1	Period 2	Period 3	Period 4	Cases liquid density from 2003 to 2005 (km^2^) (X10)	Cases liquid density from 2006 (km^2^) (X10)
Corumbê	159	69	38	46	6	22.1	12.4
Cabral	111	58	20	31	2	4.7	0
Sapinhatuba	95	11	18	57	9	71.3	13.4
Corisco	89	57	15	17	0	13.1	0
Mamanguá Direito	88	32	41	12	3	3.9	7.0
Frade	72	19	9	36	8	4.8	3.4
Campinho	69	50	5	12	2	1.9	5.7
Paraty	62	26	9	23	4	22.0	6.0
Barra Grande	57	32	3	21	1	7.8	0
Verolme	54	21	9	19	5	1.2	0.6
Japuiba	53	19	15	17	2	2.3	1.5
Angra dos Reis	49	30	9	9	1	2.7	2.0
Taquari	42	19	6	17	0	9.8	0
Penha	38	22	5	10	1	2.0	1.5
Ponte Branca	37	16	5	16	0	17.7	0
Ponta Negra	22	13	3	2	4	0.8	0
Praia do Aventureiro	22	6	3	12	1	2.0	5.9
Abraão	21	4	2	11	4	2.2	0
Ilha do Araújo	19	8	2	7	2	12.8	0
Perequê	19	8	7	4	0	0.8	4.9
Ilha do Algodão	12	7	4	1	0	0	0
Olaria	11	2	2	7	0	0	0
Trindade	11	6	2	3	0	1.1	0
Zungú	11	0	0	11	0	0.2	7.1
São Gonçalo	8	4	1	3	0	1.2	0
Praia Vermelha	6	3	1	2	0	0	0
Indaiatuba	3	0	0	3	0	2.8	0
Praia do Sono	3	0	0	3	0	1.2	0
Condado	2	0	0	2	0	5.3	0
Tarituba	2	1	1	0	0	0	0
Vila Residencial	2	0	0	2	0	40.8	0
Sertão do Perequê	1	0	0	1	0	1.2	0
Aldeia Indígena	0	0	0	0	0	0	0
Baía da Ilha Grande	1,250	543	235	417	55	3.2	2.2

Source: Notification Increase Information System.

An environmental indicator on deforestation (vegetation reduction percentage), from a TM sensor image, bands 5.4 and 3 of the Landsat 5 satellite from 1986, and another ETM+ sensor image, bands 5, 4, and 3 of the Landsat 7 satellite from 2002. The images were orthorectified, segmented and rated by the Spring 5.0 application. We did the segmentation by grouping pixels in regions according to the spectral similarity criteria. The rating resulted in regions grouped into three classes: building area, human occupation area, and dense vegetation area. The latter included areas defined as environmental preservation areas. This rating was vectored and imported into the application TerraView 4.2.2, which made edits to correct rating errors. The rating’s aim was to identify building areas and human occupation areas we could use when calculating the liquid density of disease outbreaks. The rating of the dense vegetation area was useful when analyzing the vegetation decrease indicator^2,8^.

To check for possible errors in the scenario rating, we identified geotagged points in the field representing the three classes through GPS. We calculated the area of each class and the percentage of deforestation for each LSU.

For the LSU level, we used the Kernel estimator of TL cases, with a 3 km radius, defined based on the average distance between the LSU and the quartic function. This tracing helped define the areas of greater production intensity of disease cases. The areas identified by the Kernel were overlaid with indicators: percentage of households connected to the main water network (REGERAGUA), percentage of responsible individuals with a minimum wage income (RESPREND), and percentage of population increment from 1991 to 2000 (Increment) to characterize these territorial sub-municipal units.

For temporal analysis, we prepared a chart with the five LSU with greater case production from 1990 to 2006.

The LSU with greater cases concentration at the beginning of the studied series (1990), Paraty and Corisco, were marked to indicate the movement of the epidemic-enzootic process of TL in the examined region. For each following year, we identified LSU through trend analysis, i.e., the units who had a number of cases greater than five and an increase when compared to the previous year, seeking to differentiate the beginning of an epidemic outbreak and the occurrence of sporadic cases. Arrows, connecting the main LSU who were part of the process, indicated the path and the direction of the epidemic-enzootic process.

The Escola Nacional de Saúde Pública Sérgio Arouca’s Research Ethics Committee of the Oswaldo Cruz Foundation (Protocol 68/05 on July 1, 2005) approved this study.

## RESULTS

**Figure 2:**
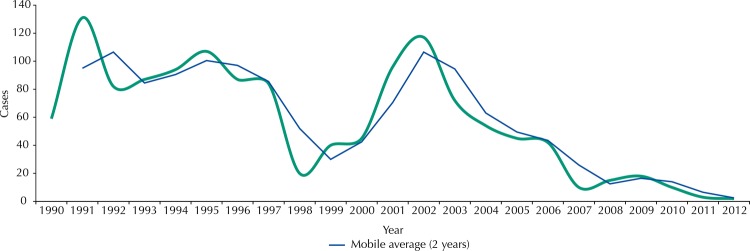
Distribution of tegumentary leishmaniasis cases. Baía da Ilha Grande region, State of Rio de Janeiro, Brazil, from 1990 to 2012.

We noticed 1,250 cases of TL in three periods up to 2006, due to the magnitude of the occurrence of disease cases (Figure 2). The largest number of TL cases (543 notifications) occurred in the first period, from 1990 to 1995. The second, from 1996 to 2000, was a period of transmission decline, although 235 cases of the disease were recorded, and in the third, from 2001 to 2006, there was a resurgence in notifications, with 417 new cases. The years 2007 to 2012 were excluded from the analysis by the occurrence of sporadic cases in the region (55 notices).

Five LSU stood out for the intensity of TL transmission in each period: Corumbê, Cabral, Sapinhatuba, Corisco, and Mamanguá Direito (Table).

When assessing the risk of transmission in the locales in 2006 compared to the average of the previous three years (2003 to 2005), we can see the disease’s instability at a sub-municipal level. Some LSU with a large liquid density average of cases from 2003 to 2005, such as Sapinhatuba with 71.2 cases per 10 km^2^ and Corumbê with 22.1 cases, had a decrease in liquid density in 2006, with 13.4 and 12.4 cases per 10 km^2^, respectively. Some LSU managed to interrupt the transmission this past year, such as Cabral and Corisco. Some LSU with a low liquid density average of cases from 2003 to 2005, such as Mamanguá Direito with 3.9 cases per 10 km^2^ and Campinho with 1.9 cases, had an increase in liquid density in 2006, representing 7.0 and 5.7 cases per 10 km^2^, respectively (Table).

**Figure 3:**
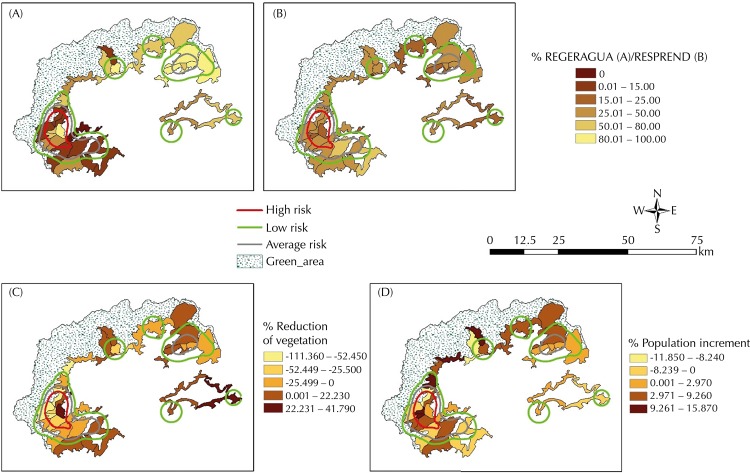
Areas with greater intensity of tegumentary leishmaniasis cases and indicators per local surveillance unit. Baía da Ilha Grande region, State of Rio de Janeiro, Brazil, from 1991 to 2000.

The LSU of Paraty, except the headquarters, seemed to be in greater need in regards to houses connected to the main water network and in regards to the main income of such households (Figures 3, A, and B). We identified an important occurrence of cases in LSU with good values in those indicators in areas with greater TL case intensity and in those with not as great values. This indicates that the economic situation was not determinant for the occurrence of disease cases in the region.

The area of greater intensity of cases comprises a central LSU (20 – Paraty) which features a great reduction in vegetation, although its close LSU neighbors border with environmental preservation areas that maintained their green area and others with an increase in vegetation, as well as borders with areas of environmental preservation (Figure 3, C). We observed no differences in a populational increase in areas of intensive TL cases (Figure 3, D). It can occur both in areas with population increase and in areas with population decrease.

REGERAGUA: Percentage of houses connected to the main water network.

RESPREND: Percentage of responsible individuals with minimum wage or lower income.

**Figure 4:**
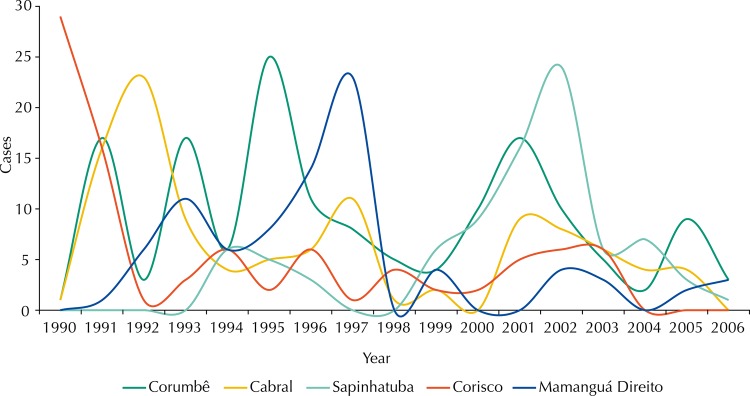
Distribution of tegumentary leishmaniasis cases in the five local surveillance units with greater occurrence of cases. Baía da Ilha Grande region, State of Rio de Janeiro, Brazil, from 1990 to 2006.

The temporal dispersion analysis (Figure 4) in the five LSU (Corumbê, Cabral, Spinhatuba, Corisco, and Mamanguá Direito) with higher production of TL cases from 1990 to 2006, showed a great variance in aggravation between the units, the continuity of transmission in the region and non-synchronicity of the epidemic process in more relevant LSU. This suggests a shift in the process of transmission between the foci of higher activity.

**Figure 5:**
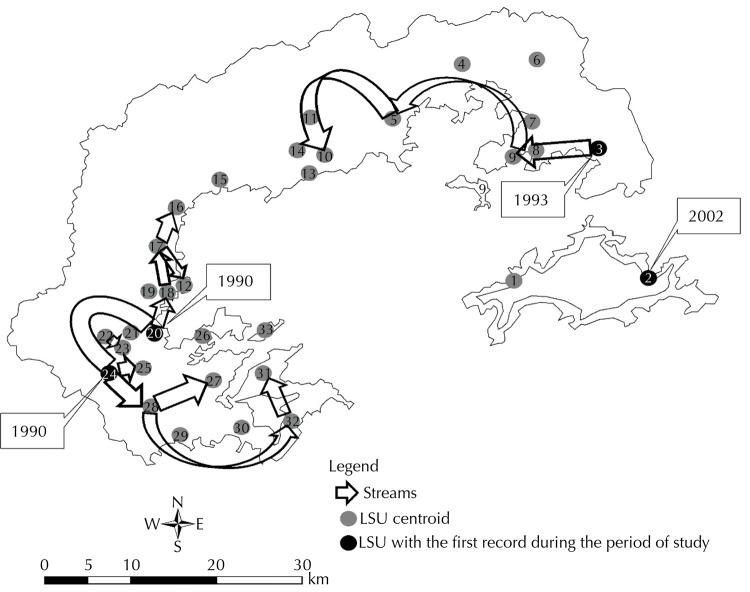
Main streams of tegumentary leishmaniasis per local surveillance unit (LSU). Baía da Ilha Grande region, State of Rio de Janeiro, Brazil, from 1990 to 2006.

We identified an initial dispersal center of TL, comprised the Paraty and Corisco LSU, and three paths of greater relevance during the period: from Paraty to Taquari (1990 to 1993), from Corisco to Mamanguá (1990 to 1993), from Verolme to Perequê (1993 to 1995) and an isolated focus in Abraão LSU in 2002 (Figure 5), which had already reported the disease in 1970^1^.

## DISCUSSION

The main finding of this study was the construction of a new operational unit for analysis at a local level, which was instrumental in examining the TL’s endemic-epidemic process. That is because the ones available in the database were not completely compatible. The LSU can be the operational unit for scheduling transmission control actions, both for antivectorial control and for reservoir control.

A classical methodology used in endemic diseases surveillance and control scheduling is territorial stratification. However, at any level of study, it is not enough for an endemic-epidemic process in constant motion, such as LT. Regardless of the determinants and biological cycles involved^11^, the territorial stratification attempt will be made using data related to events that already occurred and that do not allow, at a location-level^9^, safe predictions needed for the ever changing control. Thus, a dynamic process will require an information system permanently active with upgraded monitoring.

The pattern of infection for the area of study is not one where people are wandering in the woods. Neither is it a pattern in which the vector is coming directly from the woods to the domicile, but it has characteristic of transition areas occupied by humans, by vectors and, eventually, by the synanthropic or wild reservoirs, that approach these transition areas in search of food and water, as noted by Carvalho^j^ (1993). The importance of dogs as a reservoir does not seem to support himself^k^. In areas where most of the human population has not been infected yet, transmission to humans is not lasting, even when the dogs remain infected.

On the participation of wild animals, two movements may be occurring in the ecotone: the arrival of rodents directly from the forest in search of food and water, bringing the parasite, and the infection to synanthropic rodents^4,7,l^, amplifying the transmission in peridomestic habitats. Besides humans, dogs horses and other domestic animals are infected.

The pattern of spatial and temporal distribution of human cases suggests that the changing epidemic-enzootic occurs due to population displacements of wild reservoirs towards homes in search of food and water, because of the environmental characteristics of the Atlantic Forest region. It is possible that the researchers did not find infected rodents in previous studies^16^ because they used low sensitivity procedures and because captures happened in the periods after the transmission was over. Those rodents in peridomestic habitats would not remain permanently infected. Studies by Brandão Filho et al.^6^, Afonso et al.^1^, Andrade^3^, and Azeredo-Coutinho et al.^5^ show the importance of infection in wild and synanthropic rodents in regions of the Atlantic Forest.

The substantial reduction of TL transmission, starting in 2006, suggests a depletion of susceptible individuals. This happens due to a large number of infected people in the period under examination or to the great changes observed in the territory arising from the substitution of a characteristic pattern of small communities dedicated to fishing, banana, and cassava, for another pattern, dominated by private areas used primarily for leisure and real estate speculation. The areas’ responsiveness to TL certainly decreased, resulting in a decline of vector and reservoir populations in the altered ecosystem. Although there has been a reduction in receptivity in these areas, the occurrence of sporadic cases indicates the permanence of an epidemiological structure suitable for transmission^17,18^.

The region is an unidentified epidemiological unit, and should be operated from a surveillance and control point of view^15^, even though the control must be focused. These regions, which are a set of LSU, are more stable than the localities that compose it. They allow a control operation that cannot focus only in locations with many cases, but in a certain risk area, because they have the same characteristics as those already affected.

There were some problems found in the creation of LSU, including the compatibility of territorial bases of IBGE’s rural census sector in the different census, and without it, it is impossible to build any kind of population series. However, the territorial basis of the census sector with the largest area (1991) solved this issue.

The epidemiological information concerning places of LT transmission had a problem related to the difficulty characterizing the population exposed to risk. Although for focal diseases, the most elementary unit of analysis is the focus, population information for that level is not available in the system. Even considering the LSU as the smallest unit of sub-municipal analysis, most of the population residing in these areas is not exposed to the risk of acquiring LT. In addition, the change in population between LSU can be great, showing completely different rates in a single case. In this situation, the incidence or detection coefficients do not express the strength of transmission but reflect mainly the proportion of exposed individuals. To try to reduce this problem, we used the liquid density of TL cases.

Studies on the dynamics of the diffusion process are necessary, using quantitative models of streams to define trajectories that are more sensible and predict the locations of TL transmission inside an endemic area. The transmission process suggests the presence of reservoirs moving continuously in the region and we can hardly explain their dispersal pattern simply by the transportation of people, dogs, or infected vectors, because, at the local level, the epidemic outbreaks are always transient and short-lived.

## Footnotes

a Valim C. Transmissão da *Leishmania (Viannia) brasiliensis* no Ceará [dissertation]. Rio de Janeiro (RJ): Escola Nacional de Saúde Pública da Fundação Oswaldo Cruz; 1993.

b Sabroza PC. O domicílio como fator de risco na leishmaniose tegumentar americana: estudo epidemiológico em Jacarepaguá, município do Rio de Janeiro [dissertation]. Rio de Janeiro (RJ): Escola Nacional de Saúde Pública da Fundação Oswaldo Cruz; 1981.

c Fundação Nacional de Saúde. Vigilância e monitoramento da leishmaniose tegumentar no Brasil: relatório da reunião técnica do programa da leishmaniose tegumentar. Brasília (DF); 2003.

d Instituto Chico Mendes de Conservação da Biodiversidade. Área de Proteção Ambiental Cairuçu: nossa história: as estradas e o turismo. Paraty (RJ): ICMBio; 2005 [cited 2015 Jul]. Available from: http://www.icmbio.gov.br/cairucu/quem-somos/nossa-historia.html?start=2

e Instituto Brasileiro de Geografia e Estatística. Censo demográfico, 1970. Rio de Janeiro; 1970. p.152 e 157 [cited 2015 Jun]. Available from: http://biblioteca.ibge.gov.br/visualizacao/periodicos/69/cd_1970_v1_t16_rj.pdf

f Instituto Brasileiro de Geografia e Estatística. Censo Demográfico, 2010: resultados do universo agregados por setores censitários. [cited 2015 Jun]. Available from: ftp://ftp.ibge.gov.br/Censos/Censo_Demografico_2010/Resultados_do_Universo/Agregados_por_Setores_Censitarios/

g Soares VB. Proposta de vigilância epidemiológica da leishmaniose tegumentar em nível local: análise de indicadores para região endêmica da mata atlântica no estado do Rio de Janeiro de 1990 a 2004 [dissertation]. Rio de Janeiro (RJ): Escola Nacional de Saúde Pública da Fundação Oswaldo Cruz; 2006.

h Instituto Brasileiro de Geografia e Estatística. Malha digital de setores censitários, 1991. Rio de Janeiro. CD do IBGE com dados do Censo Demográfico de 1991.

i Instituto Brasileiro de Geografia e Estatística. Geociências, 2000. Rio de Janeiro: malha digital de setores censitários. [cited 2017 Feb]. Available from: http://downloads.ibge.gov.br/downloads_geociencias.htm

j Carvalho RW. Levantamento de aspectos da fauna de flebotoníneos (Díptera, Phlebotomine) na Ilha do Araújo, Município de Parati Estado do Rio de Janeiro [dissertation]. Rio de Janeiro (RJ): Universidade Federal Rural do Rio de Janeiro, 1993.

k Ministério da Saúde (BR), Secretaria de Vigilância em Saúde, Departamento de Vigilância Epidemiológica. Manual de vigilância da leishmaniose tegumentar americana. 2.ed. atual. Brasília (DF): Editora do Ministério da Saúde; 2013 [cited 2015 Aug 15]. Available from: http://bvsms.saude.gov.br/bvs/publicacoes/manual_vigilancia_leishmaniose_tegumentar_americana_2edicao.pdf

l Tolezano JE, Taniguchi HH, Bisugo MC, Araújo MFL, Cunha EA, Elias CR, Larosa R. Occurrence of natural *Leishmania* (*Leishmania*) infection in *Proechimys heringi* in an endemic area of human and canine American cutaneous leishmaniasis due to *Leishmania* (*Viannia*) *braziliensis* in Ilhabela, São Paulo, Brasil. In: Resumos da XII Jornada Paulista de Parasitologia da Sociedade Brasileira de Parasitologia, II Jornada Educacional e Biomédica da Superintendência de Controle de Endemias; 1998; Taubaté, SP. p. 9.
